# Psychogeriatrics and case-mix in residential and home services

**DOI:** 10.1192/j.eurpsy.2021.1143

**Published:** 2021-08-13

**Authors:** A. Riolo, U. Albert

**Affiliations:** 1 Department Of Mental Health, ASUGI, Trieste, Italy; 2 Department Of Mental Health, University of Trieste, Trieste, Italy

**Keywords:** Psychogeriatrics, Case-mix, multimorbidity, Long Term Care

## Abstract

**Introduction:**

The frail elderly with multimorbidity and polytherapy may need both residential and home services. The psychogeriatric patient can make both of these contexts very demanding and painful, so that the care burden increases.Psycho-behavioral events lead to an unexpected and particularly complex workload, requiring specific and integrated skills in the fields of health, social assistance and education.

**Objectives:**

Evaluate whether the integrated team, operating in the health district, is able to intecept multimorbidity in the presence of psychogeriatric disorders. A possible index of the ability to take charge of psychogeriatric multimorbidity is to measure admission rates to acute psychiatric services or to nursing homes.

**Methods:**

Metodi. This is an observational study on a cohort of thirty elderly patients over-65, consecutively assessed in the health district with multimorbidity and psycho-behavioral, followed for six months.

**Results:**

One third of psychogeriatric patients with multimorbidity, despite being intercepted by health services of community, are admitted to acute psychiatric services for brief observation or hospitalization. Psychogeriatric patients have high clinical instability, reducing ability to make adequate choices, lower levels of consistent actions.

**Conclusions:**

Organizational models, in response to the growing multimorbidity, and the allocation of resources cannot be oriented to the single pathology but to groups of patients in the perspective of long term care. The case-mix is an index of the complexity of the cases treated; when we refer to the psychogeriatric population, this index is high, due to emergence of social and medical problems in both residential and home services.

